# Single-Step GBLUP and GWAS Analyses Suggests Implementation of Unweighted Two Trait Approach for Heat Stress in Swine

**DOI:** 10.3390/ani12030388

**Published:** 2022-02-05

**Authors:** Gabriella Roby Dodd, Kent Gray, Yijian Huang, Breno Fragomeni

**Affiliations:** 1 Animal Science Department, University of Connecticut, Storrs, CT 06269, USA; gabriella.dodd@uconn.edu; 2 Smithfield Premium Genetics, Rose Hill, NC 28458, USA; kgray@smithfield.com (K.G.); yhuang@smithfield.com (Y.H.); 3 Institute for System Genomics, University of Connecticut, Storrs, CT 06269, USA

**Keywords:** genomic selection, genotype × environment interaction, genome-wide association study, gene identification, QTL, ssGBLUP

## Abstract

**Simple Summary:**

A major problem in the swine meat industry is significant weight loss due to pigs overheating. Our approach to solving this problem is breeding swine based on their ability to survive in high temperatures. We determined in this study that genetically there is a difference between the most successful animal in hot conditions and the most successful animal in cooler conditions. This can be applied to selection of breeding animals for swine companies. Additionally, we found a few genes that may be related to this heat resistance, but more studies need to be completed to validate this finding.

**Abstract:**

The purpose of this study was to perform a genome-wide association study to determine the genomic regions associated with heat stress tolerance in swine. Phenotypic information on carcass weight was available for 227,043 individuals from commercial farms in North Carolina and Missouri, U.S. Individuals were from a commercial cross of a Duroc sire and a dam resulting from a Landrace and Large White cross. Genotypic information was available for 8232 animals with 33,581 SNPs. The pedigree file contained a total of 553,448 animals. A threshold of 78 on the Temperature Humidity Index (THI) was used to signify heat stress. A two-trait analysis was used with the phenotypes heat stress (Trait One) and non-heat stress (Trait Two). Variance components were calculated via AIREML and breeding values were calculated using single step GBLUP (ssGBLUP). The heritability for Traits One and Two were calculated at 0.25 and 0.20, respectively, and the genetic correlation was calculated as 0.63. Validation was calculated for 163 genotyped sires with progeny in the last generation. The benchmark was the GEBV with complete data, and the accuracy was determined as the correlation between the GEBV of the reduced and complete data for the validation sires. Weighted ssGBLUP did not increase the accuracies. Both methods showed a maximum accuracy of 0.32 for Trait One and 0.54 for Trait Two. Manhattan Plots for Trait One, Trait Two, and the difference between the two were created from the results of the two-trait analysis. Windows explaining more than 0.8% of the genetic variance were isolated. Chromosomes 1 and 14 showed peaks in the difference between the two traits. The genetic correlation suggests a different mechanism for Hot Carcass Weight under heat stress. The GWAS results show that both traits are highly polygenic, with only a few genomic regions explaining more than 1% of variance.

## 1. Introduction

Heat stress is a problem for almost all livestock producers around the world. However, swine are disproportionately sensitive to high temperatures because they are unable to sweat. In response to high temperatures, swine have been shown to experience weight loss due to reduced feed intake and physiological changes [1]. This weight loss has significant impacts on both breeding and finishing herds in the swine industry. The economic loss due to heat stress is estimated to be over $299 million each year in the United States alone, with $202 million of that economic loss coming from finishing herds [2]. Many breeding programs use purebred animals from nucleus farms, however previous studies have demonstrated that crossbred animals have more significant impacts from heat stress [3]. Because of this difference in heat stress impact, selection programs based on data from purebred animals has proven ineffective [4]. An efficient alternative to this approach is to use data from commercial animals when developing a heat stress tolerance breeding program.

Genetic evaluations of heat stress have been assessed utilizing multiple models for calculation, including multiple-trait and random regression models. Zumbach et al. performed both a two-trait and a random regression analysis of heat stress in crossbred swine in 2008 [5]. The two-trait method consists of splitting the phenotypes in two based on the heat load, if the genetic correlation indicates genotype-by-environment interaction, such approach can be used to model heat stress. Both models showed evidence of genotype × environment interaction due to heat stress. Each model was later extended for inclusion of genomic information under a heat stress reaction norm in a similar population which confirmed the findings [6]. Moreover, this study established a pipeline for genomic evaluation for heat stress using single-step GBLUP (ssGBLUP) [7,8]. The ssGBLUP approach allowed for incorporation of weather data into genomic evaluation without changing existing methodology for studying the genetics of heat stress as it allows the use of multiple-trait models and reaction norms with little change to the existing software [9].

The incorporation of genomic data has the ability to increase accuracy of prediction for young animals [10]. Moreover, the use of dense SNP chips can help in the identification, detection, and mapping of genes and QTL using genome-wide association studies (GWAS) [11]. Additionally, GWAS results can be used to reconstruct the genomic relationship matrix and may increase the accuracy of prediction depending on the genetic architecture of the trait [12,13]. The main limitation for GWAS in the Fragomeni et al. study on heat stress tolerance was that results from a random regression model may be difficult to interpret and modifications must be made to Manhattan Plots for ease of interpreting GWAS results [6].

This study will attempt to establish a useable method for heat stress resistance in swine breeding programs. The primary objective of this study is to utilize a two-trait animal model analysis to evaluate the efficacy of selecting for heat stress resistant pigs. Additionally, we will identify the SNP windows that may play a role in swine ability or inability to regulate temperature.

## 2. Materials and Methods

Animal Care and Use Committee approval was not obtained for this study because data were obtained from an existing database.

### 2.1. Data

Data were collected at packing plants in North Carolina and Missouri on a commercial cross of a Duroc sire and a dam resulting from a Landrace and Large White cross. All data were collected within a single company which maintained consistent management practices for feed and housing. Phenotypes were measured as Hot Carcass Weight (HCW) at an average slaughter age of 185 days. Phenotype data were collected between August 2008 and July 2014. There was overlap between the two farms with approximately 30% of sires having progeny in both states. Descriptive statistics for the study population are given in Table 1.

Cleaning of the data set consisted of removing animals missing both parents, animals with pedigree conflicts, and any outliers with records above 4 standard deviations from the mean. The pedigree file contained a total of 553,448 animals after cleaning. Of this group, 227,043 animals had records and 8232 had genotypic information available. Genotype data is from the Infinium PorcineSNP60 v2 BeadChip (Illumina, Inc., San Diego, CA, USA). Genotypes were imputed following the same procedures as Fragomeni et al. [6]. Quality Control (QC) was also conducted prior to the start of this study and removed Single Nucleotide Polymorphisms (SNPs) with minor allele frequency less than 0.05 and call rates less than 0.9, monomorphic SNP, and genotypes with call rates less than 0.9. After QC, we were left with 33,581 SNP.

Weather data was collected using the R package weatherData [14] using airport weather stations near the farms as reference points. In a previous study performed by Fragomeni et al., it was determined that the difference in the 30-day heat load between weather stations located closer to the farms and weather stations located at nearby airports was insignificant (r=0.99) [6]. For North Carolina farms, the Wilmington airport was used for data collection due to the completeness of this dataset. For Missouri farms, the Des Moines airport in Iowa was the closest station. All recordings of maximum temperature and minimum humidity during the study period were used to calculate the Temperature Humidity Index (THI). The THI was calculated using the following formula:

THI = (1.8 × *t* + 32) − [0.55 − (0.0055 × *rh*)] × (1.8 × *t* − 26), (1)

where *t* was the maximum temperature in degrees Celsius and *rh* was the minimum relative humidity on a scale of 0 to 100. A regression analysis was run prior to the start of this study to determine the threshold for heat stress. A THI value of 78 was the threshold used for this study to separate heat stress and thermo-normative conditions based on convergence and fit of the model established in a previous study done by Fragomeni et al. [6]. This level of THI indicates a range from 25.5 °C with 100% humidity to 40 °C with 0% humidity. Trait One was HCW (body weight at slaughter) under heat stress which was defined as an average THI above 78 for 30 days prior to slaughter. Trait Two was HCW (body weight at slaughter) under non-heat stress which was defined as an average THI below 78 for 30 days prior to slaughter. North Carolina had a higher proportion of phenotype collection in Trait One when compared to Missouri.

### 2.2. Model and Analysis

Genetic analysis was performed using a two-trait animal model:

(2) $$y_{ijkl}=CG_{i}+sex_{j}+litter_{k}+b\times age+animal_{l}+e_{ijkl},$$

where $y_{ijkl}$ is the observed phenotype of animal l in Contemporary Group i of sex j in litter k; b is the regression coefficient for age, which refers to the age of the animal at slaughter (in days); animal is the random additive genetic random effect of the animal. Finally, $e_{ijkl}$ is the random residual effect. Contemporary Group was a combination of year, week, farm; litter was a combination of dam and parity. This model can also be written in matrix notation as:

(3) y=Xb+Wl+Za+e,

where y is a vector of the phenotypes, X is the incidence matrix of the fixed effects in vector b (contemporary group, gender and age); W is the incidence matrix of the random litter effects contained in l; Z is the incidence matrix of random animal effects contained in a; and e is a vector of the random residuals. Variances for the two-trait model were:

(4) $$var\left[\begin{array}{c} l_{HS}\\ l_{NHS} \end{array}\right]=I⨂\left[\begin{array}{cc} \sigma_{l_{HS}}^{2} & 0\\ 0 & \sigma_{l_{NHS}}^{2} \end{array}\right],$$

(5) $$var\left[\begin{array}{c} a_{HS}\\ a_{NHS} \end{array}\right]=A⨂\left[\begin{array}{cc} \sigma_{a_{HS}}^{2} & \sigma_{a_{HS,NHS}}\\ \sigma_{a_{HS,NHS}} & \sigma_{a_{NHS}}^{2} \end{array}\right],\;\mathrm{and}$$

(6) $$var\left[\begin{array}{c} e_{HS}\\ e_{NHS} \end{array}\right]=I⨂\left[\begin{array}{cc} \sigma_{e_{HS}}^{2} & 0\\ 0 & \sigma_{e_{NHS}}^{2} \end{array}\right],$$

where $\sigma_{j_{HS}}^{2},\;\sigma_{j_{NHS}}^{2}$ are variances of the jth effect of litter (l), additive genetic (a), and residual effect (e) for a trait under heat stress, a trait not under heat stress respectively, and $\sigma_{j_{HS,\;NHS}}^{2}$is the covariance of the jth effect between the two traits. Additionally, A is the numerator relationship matrix for all the animals in the pedigree and I is the identity matrix with dimensions equal to the number of animals with records. Narrow sense heritability for the heat stress trait was calculated as $\frac{\sigma_{a_{HS}}^{2}}{\sigma_{a_{HS}}^{2}+\sigma_{l_{HS}}^{2}+\sigma_{e_{HS}}^{2}}$, for the non heat stress trait, the same formula was used. Genetic correlations were estimated as $\frac{\sigma_{a_{HS,NHS}}}{\sqrt{\sigma_{a_{HS}}^{2}\times \sigma_{a_{NHS}}^{2}}}$.

In this model, a is a vector of the additive genetic effects which is distributed normally,

(7) $$a\sim N\left(0,A\sigma_{a}^{2}\right),$$

To include genomic information into the analysis, a ssGBLUP approach was utilized [7,8]. Such approach consists in replacing the numerator relationship matrix with a relationship matrix including full pedigree and genomic information, H [15].

The inverse of the matrix H is easily calculated using the genomic and numerator relationship matrices:

(8) $$H^{-1}=A^{-1}+\left[\begin{array}{cc} 0 & 0\\ 0 & G^{-1}-A_{22}^{-1} \end{array}\right],$$

Here, G is calculated [16]

(9) $$G=\frac{MDM^{\prime }}{2\sum_{n}^{t}p_{n}q_{n}},$$

where M is a centered matrix of SNP content, D is a diagonal matrix of weights, $p_{n}$ and $q_{n}$ are gene frequencies, and t is the number of SNPs. For the initial implementation, D=I, where I is an identity matrix with dimensions equal to the number of genotyped individuals. The identity matrix approach considers that each SNP has equal contribution to the phenotypes.

In addition to the two-trait analysis, a single-trait analysis was run for both Trait One and Trait Two to compare results. The same model was adopted for the single-trait approach, and the assumptions for the variances of the random effects were the same as the two-trait approach.

### 2.3. Genome Wide Association Study

SNP effects were calculated by back solving GEBVs, following VanRaden and Zhang [16,17].

(10) $$\hat{u}=DM^{\prime }{\left[MDM^{\prime }\right]}^{-1}\hat{a},$$

where u is the vector containing SNP solutions and $\hat{a}$ is the vector with the solutions for the random additive genetic effect.

The elements of the D matrix were updated in an iterative approach, described by Wang et al. [12]. The values for the diagonal elements of D were calculated using Quadratic and Nonlinear “A” methods for estimates of the variance of individual SNPs, $\sigma_{u,s}^{2}$. Variance calculation in the Quadratic method is

(11) $$\sigma_{u,\;s}^{2}={\hat{u}}_{s}^{2},$$

where ${\hat{u}}_{i}$ is the estimated SNP effect for the *i*th marker. This approach follows the methodology from Wang et al. [12], without the allelic frequencies. Preliminary results (not shown) presented lower accuracies when $2p_{i}q_{i}$ were multiplied by ${\hat{u}}_{i}^{2}$. Variance calculation in the Nonlinear “A” method is

(12) $$\sigma_{u,\;s}^{2}=\frac{\sigma_{a}^{2}}{2\sum_{n=1}^{t}p_{n}q_{n}}1.25^{\frac{\left|{\hat{u}}_{s}\right|}{sd\left(\hat{u}\right)}-2},$$

which is from methods by VanRaden based on BayesA [16]. In this method, $\left|{\hat{u}}_{s}\right|$ is the absolute value of estimated SNP effect for marker s and $sd\left(\hat{u}\right)$ is the standard deviation of the estimated SNP effects. The value of 1.125 was a constant representing departure from normality. Additionally, the exponent was limited at 5, to avoid extreme values [18].

GWAS results were compiled in Manhattan Plots including the position of the SNPs windows within chromosomes and the percent of the total additive genetic variance explained by that window. Moving windows containing 20 markers each were calculated all over the genome based on the average variance of the SNPs in that window. The SNP variance of a SNP s was calculated as ${\hat{\sigma }}_{u,\;s}^{2}={\hat{u}}_{s}^{2}\;2p_{s}\left(1-p_{s}\right)$, where $p_{s}$ is the major allelic frequency of that SNP [16,17]. Upon completion of the GWAS analysis, a threshold of 0.8% was selected based on visual inspection of the Manhattan Plots. This threshold was established to separate SNP peaks that were investigated for biological significance from those that were considered to have a relatively low level of variance explanation. Establishing a threshold was necessary due to the polygenic nature of this trait. Peaks that were considered noteworthy were studied to determine whether there is any literature or documentation on genes and QTLs located in or near these regions. Sear parameters allowed for gene or QTL results within 5000 bp upstream or downstream from SNPs above a threshold of 0.8% of the variance explained in the population. Specific QTLs were additionally located manually using a comprehensive database of Swine QTLs with current annotations on the genome.

Genetics of thermotolerance was assessed as the difference between the additive genetic effect below or above the threshold for the heat load. However, the single-step GWAS generates a single Manhattan Plot for each trait, which makes interpretation of the results difficult. Therefore, a third Manhattan Plot was generated by plotting the difference in the variance explained by each region in the two traits. The working hypothesis for this approach is that genomic regions associated with thermotolerance will have different effects under normal and heat stress conditions.

### 2.4. Validation and Accuracy

For validation calculations, estimated breeding values were calculated both with a reduced and complete dataset. The reduced dataset excluded records collected after 1 October 2013. Validation was calculated for 163 sires with at least 200 progeny with records in the complete data, but no progeny in the reduced data.

Accuracy estimates of each model were evaluated using the correlation between the estimated breeding value (EBV) and the “true” breeding value (TBV). The EBVs were calculated for the validation boars in each SNP weighting method with the reduced data, and the hereby termed TBV were calculated using the two-trait unweighted ssGBLUP with the complete data, following the validation performed by Fragomeni et al. and the LR method [6,19]. Accuracy was first calculated by using the correlation between Trait One TBV with Trait One EBV, termed homo-correlation. Accuracy was then calculated by using the correlation between Trait One TBV and Trait Two EBV, termed hetero-correlation. Accuracy was calculated for each combination of Trait One and Trait Two results for EBV and TBV. The total number of accuracy calculations was four.

### 2.5. Software

To estimate variance components, an average information restricted maximum likelihood (AIREML) method was used. Calculation of the variance components was done utilizing the AIREMLF90 software version 1.149. Additionally, breeding values were predicted as the best linear unbiased predictor (BLUP) using Henderson’s mixed model equation. An ssGBLUP approach was used for genomic enhanced breeding values (GEBV). Breeding value calculations were done using the BLUPF90 program version 1.71 [20]. For the GEBV and SNP effects, preGSF90 and postGSF90 [21] version 1.23 and 1.74 respectively were used. Isolated peaks identified through the GWAS were identified through the R Package GALLO version 1.3 [22] and manual inspections using PigQTLdb [23].

## 3. Results

Heritability, genetic correlation, and variance components results are displayed in Table 2. Heritability calculations showed increased heritability for Trait One, HCW under heat stress, with a heritability of 0.25. Trait Two, HCW under normal conditions, had a slightly lower heritability calculated at 0.20. The genetic correlation between the two traits was calculated to be 0.63.

### 3.1. GWAS

**Figure 1:**
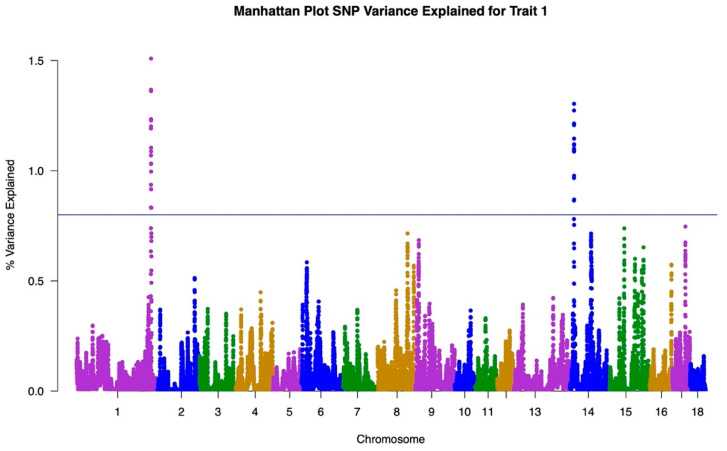
Manhattan Plot representing the Genome-Wide Association Study results for Trait One, referring to HCW under heat stress conditions. Chromosomes listed on x axis and percentage of variance explained listed on the y axis.

**Figure 2:**
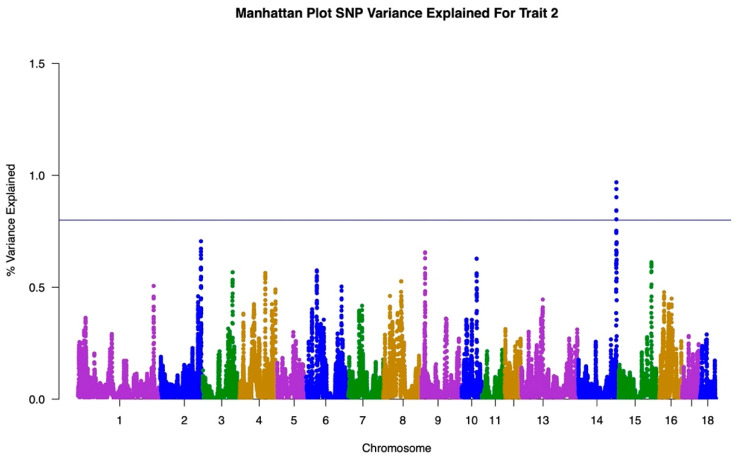
Manhattan Plot representing the Genome-Wide Association Study results for Trait Two, referring to HCW under thermos-neutral conditions. Chromosomes listed on x axis and percentage of variance explained listed on the y axis.

**Figure 3:**
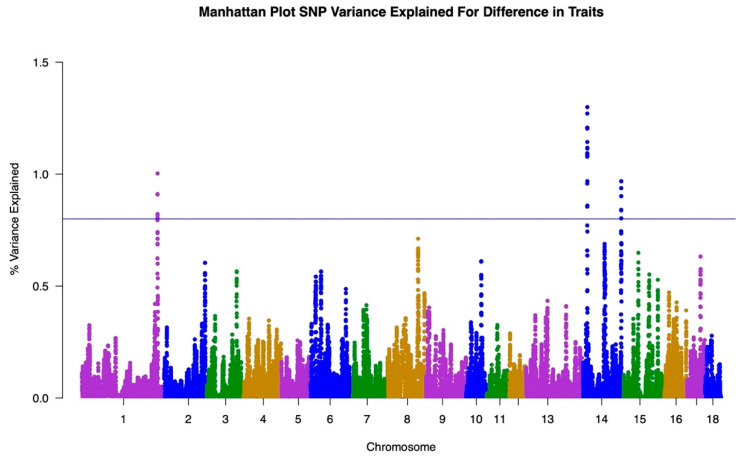
Manhattan Plot representing the difference between the Genome-Wide Association Study results for Trait One, referring to HCW under heat stress, and Trait Two, referring to HCW under normal conditions. Line placed to identify chromosomes that explain more than 0.8% of the variance in the difference between traits. Chromosomes listed on x axis and percentage of variance explained listed on the y axis.

The GWAS results for both traits are illustrated in Manhattan Plots in Figure 1, Figure 2 and Figure 3. The single-trait analysis produced similar results to the two-trait approach, therefore the single trait results are not shown. To illustrate the genetic influence of HCW under heat stress and HCW not under heat stress as separate traits, the GWAS results from the single-trait analysis for Traits One and Two were used.

Trait One, representing HCW under heat stress, is shown in the Manhattan Plot in Figure 1. Due to the polygenic nature of this trait, a threshold of 0.8% of the variance explained was established to separate SNPs with greater association and decrease the number of SNPs to be investigated for links in heat stress. Peaks above this threshold of 0.8% were analyzed. For this trait, peaks were isolated at Chromosome 1 and Chromosome 14; both explaining approximately 1.4% of the variance in the trait. The remainder of the peaks explained around 0.7% of the variance or less. The same line at 0.8% variance explained was used when analyzing the Trait Two GWAS results. Trait Two, representing HCW under normal conditions, is shown in the Manhattan Plot in Figure 2. The largest peak for Trait Two occurred at Chromosome 14, which explained approximately 1% of the variance in the trait. This is followed by Chromosome 1 which explained approximately 0.7% of the variance. The GWAS results for Trait Two consisted of fewer break-out peaks with more lower influence peaks relative to the Trait One results.

To fully demonstrate the differences and overlaps between the Trait One and Trait Two results, a Manhattan Plot for the difference between results was generated, shown in Figure 3. This plot was created by taking the absolute value of the difference between the variance explained by Traits One and Two from the single-trait analyses. This plot was used to establish SNP associations for the ability to resist heat stress conditions-heat tolerance. These results showed major peaks at Chromosomes 1 and 14. In the plot for the difference between the traits, a peak on Chromosome 1 explains approximately 1.1% of the variance and a peak on Chromosome 14 explains approximately 1.3% of the variance. The remainder of the peaks fell to 0.7% of the variance or below when the difference was taken.

Utilizing the R package Gallo [22], SNP regions with a percentage of variance explained in the population at or above 0.8% were analyzed for known genes. Multiple regions at this variance threshold in the population were determined to be protein coding regions for Centrosomal Protein 44 (CEP44), F-Box Protein 8 (FBXO8), 15-Hydroxyprostaglandin Dehydrogenase (HPGD), and Glycine Receptor Alpha 3 (GLRA3). All four of these genes were found on Chromosome 14 with CEP44 at 15,888,733 bp, FBXO8 at 15,889,768 bp, HPGD at 15,734,321 bp, and GLRA3 at 15,590,405 bp.

The manual search of Quantitative Trait Loci (QTL) in the regions at this threshold revealed two QTLs for production traits and one for health traits. An isolated peak on Chromosome 1 at 289 kbp had a variance explained of approximately 1% and was linked to body mass and growth traits. An isolated peak on Chromosome 14 at 16 kbp also explained 1% of the variance in the trait and was linked to traits involving diseases susceptibility and back fat thickness.

### 3.2. Validation and Accuracy

**Figure 4:**
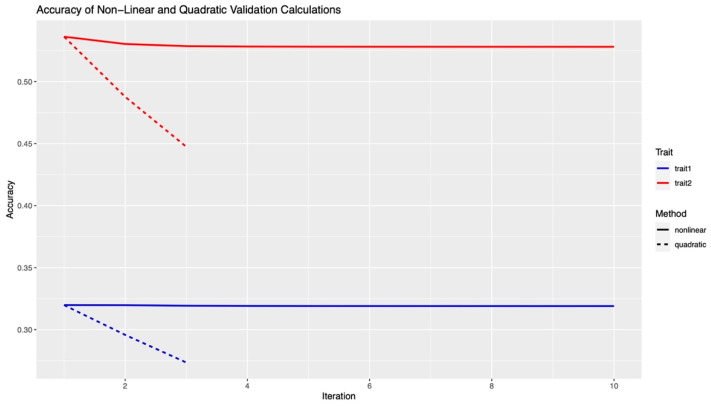
Graph showing the accuracy calculations for the Nonlinear “A” and Quadratic Methods. Blue lines represent Trait One calculations for Nonlinear “A” (solid line) and Quadratic method (dotted line). Red lines represent Trait Two calculations for Nonlinear “A” (solid line) and Quadratic method (dotted line). The x-axis shows the iteration, ten for Nonlinear “A” and three for Quadratic, and the y-axis shows the accuracy calculated by correlation.

The weighted single step GBLUP analysis was tested using the reduced set of data. Accuracy was calculated using the homo-correlation and hetero-correlation approaches mentioned above. The results showed no difference in accuracy between these two approaches for both Nonlinear and Quadratic weighting methods. Accuracy calculations for the two methods of both traits are shown in Figure 4.

Nonlinear “A” weighting methods showed a slight decrease in accuracy as SNP weights were allowed to vary. For both Trait One and Trait Two, the accuracy for the Nonlinear “A” method peaked at iteration one. The initial accuracy was calculated at 0.32 for Trait One and at 0.54 for Trait Two. For the homo-correlation calculations, Trait One showed a drop in accuracy of $1\times 10^{-4}$ from iteration one to iteration two while Trait Two showed a decrease of $6\times 10^{-3}$. For the hetero-correlation calculations, Trait One showed a drop in accuracy of $2\times 10^{-3}$ from iteration one to iteration two while Trait Two showed a decrease of $5\times 10^{-3}$). All drops in accuracy as iterations progressed were negligible for the Nonlinear “A” weighting methods.

Quadratic weighting methods showed a greater decrease in accuracy as SNP weights varied. For both Trait One and Trait Two, the accuracy for the Quadratic method peaked at iteration one as well. The initial accuracies were calculated to be the same as the Nonlinear “A” method. For both homo-correlation and hetero-correlation calculations, Quadratic method showed a greater decrease in accuracy after the first iteration than was found in the Nonlinear “A” calculations. Traits One and Two showed a drop in accuracy of 0.1 from iteration one to iteration two for both calculations. Accuracies continued to drop as iterations progressed.

## 4. Discussion

Hot Carcass Weight under heat stress had a higher heritability when compared to that without heat stress. This pattern of greater heritability at high temperatures has been found in previous studies as well [5,24]. The higher heritability calculated for Trait One supports selection for heat tolerance and suggests decreased genotype × environment interaction. Numbers calculated in this study were similar to those found in the study done by Usala et al. for various carcass traits though were lower that found by Zumbach et al. [5,24]. The smaller values for heritability relative to the Zumbach study may be due to differences between the populations used. The Zumbach et al. [5,24] study was performed with 23,556 animals born in 2005 and 2006, therefore, selection and sample size could contribute to differences. When using a random regression reaction norm approach on the same dataset, Fragomeni et al. found that higher heat loads resulted in higher heritability which matches what was found through this approach [6]. This indicates that using this simpler approach will obtain a similar trend in results and interpretation and has more practical application for industry implementation of genotype × environment analysis.

The genetic correlation in this study was found to be less than 0.70, which is the critical threshold to assume genotype × environment interaction [25]. Zumbach et al. found a genetic correlation of 0.42 between HCW under heat stress and not under heat stress with a similar population, which was less than half of that found in this study [5]. Possible explanations for this difference are the number of animals in the study, differences in the populations, different implementations of heat load, and a different threshold for separating the two traits. Robertson suggested that genetic correlations below 0.8 would result in significant sire re-ranking [26]. Conversely, Mulder et al. concluded that in some circumstances, traits with genetic correlations above 0.6 could be analyzed as a single trait [27] depending on the number of observations in each trait. Based on changes in heritability and genetic correlations in the present study, HCW under heat stress and HCW not under heat stress were determined to be separate traits it can be concluded that selection for the two traits would be separate, however it is possible to select in favor of both traits.

The GWAS results further illustrate the differences in genomic regions underlying each trait. Both traits presented as polygenic in nature, with few regions explaining more than 0.8% of the genetic variance. The differences in the peak patterns between the Trait One and Trait Two Manhattan Plots were used to identify SNP associations in heat tolerance only (Figure 3). This Manhattan Plot was created by taking the absolute value of the difference in GWAS results for Traits One and Two to isolate any genetic patterns for the trait of heat tolerance.

The GWAS results demonstrated the overall complexities of both traits for HCW. Figure 1 and Figure 2 show a wide distribution of SNPs explaining the variance in each trait indicating that both HCW under heat stress and HCW not under heat stress are polygenic. This is also supported by the results shown in Figure 3. The heat tolerance Manhattan Plot shows a few major peaks with a wide distribution of SNPs explaining less than 0.8% of the genetic variance. Heat tolerance association was represented by the difference between the Manhattan Plots for Traits One and Two. Similar to Traits One and Two, heat tolerance was shown to be polygenic. Though we are unaware of studies showing a GWAS for heat tolerance in swine, bovine studies had similar results to those found here when studying rectal temperature as a measure of ability to regulate body temperature during heat stress [28,29]. Otto et al. identified only three significant SNP while Dikmen et al. found that the major windows with three or five SNPs explained less than 0.45% of the genetic variance [28,29]. In both cases, the studies identified a complex genetic architecture for heat tolerance in bovine.

Results from the two weighted ssGBLUP analyses provided further evidence that Traits One and Two are polygenic. As SNP weights varied using Quadratic weighting methods there was a decrease in accuracy. This indicates the polygenic nature of heat stress traits as it has been shown in previous studies that Quadratic weighting will only improve accuracy when there are only a few major QTLs or genes affecting a trait [30]. There was quick convergence in accuracy as SNP weights varied using Nonlinear “A” weighting methods. This result was shown by Fragomeni et al. where it was concluded that Nonlinear “A” weighting showed a stability in accuracy in complex traits [6]. The consistent decrease in accuracy suggests that prediction is not improved by letting SNPs have greater or less weights. The added complexity of fitting multiple G matrices requires additional time and storage and is therefore not advisable given this loss of accuracy. Additionally, in commercial swine genetic evaluations multiple traits are considered, including maternal, growth, carcass, and health traits. For a multi-trait weighted genomic evaluation, such programs would require calculating a trait specific genomic relationship matrix, which would only be recommended with large gains in accuracy. Thus, the added complexity of this analysis is not worth time and storage for industry applications.

The GWAS results allowed for isolation of chromosome regions that explained different proportions of genetic variance for HCW under heat stress and HCW not under heat stress. The genes located in those regions may provide insight into the biological mechanisms involved in heat tolerance. However, changes in the effects of SNPs may also be consequence of the small number of independent chromosome segments (q). The estimated number of q is $\left(2N_{e}L\right)/\log(2N_{e}L)$ [31,32]. Using the effective population size of 55 [33] and a chromosome length of 19M [34], would mean that only 577 segments exist. This would increase the predictor error variance of SNP effects due to multicollinearity. Limited dimensionality of genomic information can be used to reduce computational power for genomic predictions [35,36,37,38]. However, it may result in changes in GWAS peaks due to variation of SNP effects when data changes [39]. For this reason, the gene associations found in this analysis are not conclusive of the biology underlying heat tolerance.

Genes isolated through genomic annotation included the protein coding regions of CEP44, FBXO8, HPGD, and GLRA3. The primary function of the CEP44 protein is to stabilize cell division at the centrosome and is highly conserved in eukaryotes [40]. This gene may be linked to the ability to maintain cell division under heat stress conditions. The FBXO8 gene is also highly conserved among eukaryotes and is involved in the ubiquitination system. The ubiquitination system is involved in most cellular processes (i.e., cell cycle and division, apoptosis, antigen processing, etc.) [41]. Once again, there could be a link between the FBXO8 gene and the ability to maintain these cellular processes under heat stress conditions. The HPGD gene is primarily involved in the metabolism of prostaglandins which are involved in many cellular processes including inflammation [42]. Inflammation is often a reaction to bodily stress or infection which could communicate its importance under heat stress conditions. Finally, GLRA3 encodes for ligand gated ion channels [43]. While these genes were the nearest annotated genes, there is not a direct link to heat stress suggesting there may be other genes in this region that have not yet been annotated. Heat stress and thermotolerance are polygenic traits that may involve a complex network of genes. Therefore, the few regions identified in the current student are not able to explain the molecular processes underlying such complex phenotypes. Moreover, the genes annotated in this study were not associated with heat stress before, illustrating the need of more research in this area.

QTL regions found through manual search revealed some interesting studies that could explain the decrease in HCW when swine undergo heat stress. The peak isolated on Chromosome 1 at 289 kbp was linked to body mass and growth and is associated with transforming growth factor, beta receptor 1 (TGFBR1). This growth factor is known to impact cell proliferation, differentiation, and apoptosis [44]. This has a possible link to the ability for individuals to maintain or lose body mass through periods of heat stress. The region on Chromosome 14 at 16 kbp revealed traits involving disease susceptibility and back fat thickness [45,46]. Waide et al. performed a genome wide analysis of piglet responses to infection and identified a genomic region associated with response to Porcine Reproductive and Respiratory Syndrome Virus (PRRSV) infection [45]. There could be a correlation between ability of individuals to resist virus infection and overall health and maintenance of body conditioning. In the same region of the porcine genome, Jiao et al. determined that this region is associated with nutrient utilization through a genome wide analysis of Duroc pigs [46]. The link between nutrient utilization and HCW is reasonably well supported through the literature [47,48,49].

These candidate genes identified through the GWAS may have an impact on phenotype or inheritance, though it is difficult to truly identify candidate genes because of the overwhelming lack on annotation in the swine genome. Using this approach may help to understand some of the underlying genetics, but it is not useful for application in breeding. For this reason, we recommend a whole genome approach for use in breeding programs.

## 5. Conclusions

This study demonstrated that HCW under heat stress and HCW not under heat stress are two different traits. This conclusion was supported first by the genetic correlation and additionally from the GWAS results. This discovery suggests that you can successfully select for heat tolerance in swine through a breeding program. Peaks were isolated at Chromosomes 1 and 14 which explained more than 1% of the variance each through the GWAS. Each region should be further investigated through functional studies. In combination with the weighted ssGBLUP, the GWAS also revealed that HCW under heat stress and not under heat stress are highly polygenic traits. The conclusions of this study hold implications for industry applications of breeding value calculations, specifically the ineffectiveness of using a weighted ssGBLUP approach for polygenic traits.

## Figures and Tables

**Table 1:** Descriptive statistics for commercial cross animals, Duroc × F1 (Landrace × Large White), for Trait One (individuals under heat stress) and Trait Two (individuals not under heat stress) and total statistics. Note: no. refers to the number of observations.

Item	Trait One: Under Heat Stress	Trait Two: No Heat Stress	Total
No.	32,783	194,260	227,043
Weight, kg	89.9	94.2	93.2
Age, d	188.10	184.87	185.30
North Carolina, no.	29,296	111,319	140,615
Missouri, no.	3487	82,941	86,428

**Table 2:** Heritability, genetic correlation, and variance components results.

Result	Trait One: Under Heat Stress	Trait Two: No Heat Stress
Random Residual Variance Component	216.69	210.69
Litter Variance Component	31.736	30.791
Genetic Variance Component	84.697	60.173
Heritability	0.25	0.20
Genetic Correlation	0.63	

## Data Availability

The phenotypic and genomic data used in this study are a property of the industry partner that contributed to the study and therefore are not readily available due to its commercially sensitivity. Requests to access these datasets should be directed to Yijian Huang, yhuang@smithfield.com.
